# High Temperature Ultrasonic Transducers: A Review

**DOI:** 10.3390/s21093200

**Published:** 2021-05-05

**Authors:** Rymantas Kazys, Vaida Vaskeliene

**Affiliations:** Ultrasound Research Institute, Kaunas University of Technology, Barsausko st. 59, LT-51368 Kaunas, Lithuania; vaida.vaskeliene@ktu.lt

**Keywords:** high temperature ultrasonic transducers, waveguide transducers, high temperature piezoelectric materials, backings, bonding technique, aluminum nitride, lithium niobate, gallium orthophosphate, bismuth titanate, oxyborate crystals, lead metaniobate

## Abstract

There are many fields such as online monitoring of manufacturing processes, non-destructive testing in nuclear plants, or corrosion rate monitoring techniques of steel pipes in which measurements must be performed at elevated temperatures. For that high temperature ultrasonic transducers are necessary. In the presented paper, a literature review on the main types of such transducers, piezoelectric materials, backings, and the bonding techniques of transducers elements suitable for high temperatures, is presented. In this review, the main focus is on ultrasonic transducers with piezoelectric elements suitable for operation at temperatures higher than of the most commercially available transducers, i.e., 150 °C. The main types of the ultrasonic transducers that are discussed are the transducers with thin protectors, which may serve as matching layers, transducers with high temperature delay lines, wedges, and waveguide type transducers. The piezoelectric materials suitable for high temperature applications such as aluminum nitride, lithium niobate, gallium orthophosphate, bismuth titanate, oxyborate crystals, lead metaniobate, and other piezoceramics are analyzed. Bonding techniques used for joining of the transducer elements such as joining with glue, soldering, brazing, dry contact, and diffusion bonding are discussed. Special attention is paid to efficient diffusion and thermo-sonic diffusion bonding techniques. Various types of backings necessary for improving a bandwidth and to obtain a short pulse response are described.

## 1. Introduction

Ultrasonic measurements and non-destructive testing (NDT) are already used in extreme conditions such as high temperature, pressure, corrosive environments, radioactive radiation, etc. Such conditions are met during online monitoring of manufacturing processes at elevated temperatures, non-destructive testing in nuclear plants, measurements and inspections in the aerospace field, etc. [1].

Ultrasonic NDT of various steel components in some cases must be performed up to 400 °C [2]. Increase of the temperature of the object under a test leads to increase of the attenuation of ultrasonic waves. Corrosion rate monitoring of steel pipes with permanently installed transducers in petrochemical refineries and nuclear plants is usually performed at elevated temperatures [2,3,4]. Ultrasonic methods which are used for online measurement of viscosity, density, and temperature during the manufacturing process of various molten plastic and glass-like materials are also done at a high 220 °C [5] or even very high temperatures reaching up to 1500 °C [6]. Ultrasonic transducers for some space applications should operate at temperatures reaching 460 °C and at the same time at high pressure of 9 MPa [7,8]. Ultrasonic methods are also used for measurements of high temperatures [9,10] and flow velocity of hot metal melts exploiting the Doppler effect [11,12].

One of the very important fields in which ultrasonic technologies for NDT and imaging are used is in nuclear plants. Quite a big number of nuclear reactors are cooled by heavy liquid metals such as sodium, lead, and lead–bismuth which are optically opaque [13,14]. The melting temperature of sodium is 97.99 °C, lead–bismuth 123.5 °C, and lead 327.5 °C; however, during operation the temperatures of coolants may be significantly higher. There were a big number of sodium cooled nuclear reactors which now are shutdown. Now there are 4 fast breeder reactors in the world which are still operating [14]. At the moment liquid sodium is used only in two Russian reactors. A shortcoming of sodium is that it is a very reactive behavior as a coolant, which affects reactor safety.

Quite a big number of fast breeder nuclear reactors cooled by sodium and lead–bismuth are planned to be built in the near future in China, India, Korea, Japan, USA, Russia, and Europe. In the European Union a few fourth generation nuclear reactors cooled by heavy liquid metals are in a process of development. The ALFRED project (Advanced Lead Fast Reactor European Demonstrator) started in the year 2010 [15]. Such a reactor possesses better features than sodium-cooled fast reactors because it exploits attractive properties of the molten lead as a coolant. It is the Lead Fast Reactor in which outlet temperatures of the coolant lead are between 400 °C and 480 °C [16].

In Russia the Industrial and Nuclear Supervision Service Rostechnadzor in the year 2021 issued a license for construction of the 300 MWe fast neutron reactor BREST-OD-300 cooled with a lead-coolant [17,18]. They claim that the necessary regulations for safe operation of the reactor and pipelines were developed. The project should be completed in 2026.

Another European project is MYRRHA (Multi-purpose hYbrid Research Reactor for High-tech Applications) project during which the accelerator driven system is built in the Belgian Nuclear Research Centre SCK•CEN at Mol [19,20]. It should demonstrate the feasibility of high-level nuclear waste transmutation at an industrial scale. The system is cooled by lead–bismuth eutectic. For licensing and secure operation two types of ultrasonic imaging and NDT techniques will be used [21]. One technique should provide images of the reactor interior filled with opaque lead–bismuth alloy and another technique will be used for NDT of reactor walls with access from outside.

Both types of reactors require ultrasonic non-destructive and imaging techniques for licensing and routine procedures for ensuring safety of reactors. As an example, the European project PASCAL can be mentioned because it is devoted to support the ongoing pre-licensing processes of fourth generation heavy liquid metal cooled nuclear reactors ALFRED and MYRRHA [22].

Application of ultrasonic techniques for such conditions requires ultrasonic transducers suitable for operation not only at elevated temperatures but also at high levels of gamma ray dose and neutron fluence, high pressure, and is resistant to corrosion [23,24,25].

The temperatures at which ultrasonic transducers with piezoelectric elements may operate are limited by the Curie temperatures of the piezoelectric elements used. The maximal operation temperature is also limited by material properties used for bonding of the transducer’s components [21].

In this review we shall pay our attention to ultrasonic transducers with piezoelectric elements suitable for operation at temperatures higher than of the most commercially available transducers, i.e., 150 °C.

The paper is organized as follows: In Section 2, the main four types of high temperature ultrasonic transducers are discussed. In Section 3, properties of currently used high temperature piezoelectric materials are analyzed. Section 4 is devoted to description of various techniques used for bonding of different elements of ultrasonic transducers and suitable for operation at high temperatures. In Section 5, backing elements for elevated temperatures are discussed.

## 2. Types of the Ultrasonic Transducers

Ultrasonic transducers used in hazardous conditions such as a high temperature, a corrosive environment and a radioactive radiation can be classified into three main types:

1. Ultrasonic transducers with a thin protective layer;
2. Ultrasonic transducers with a delay line;
3. Ultrasonic transducers with a waveguide.

**Figure 1:**
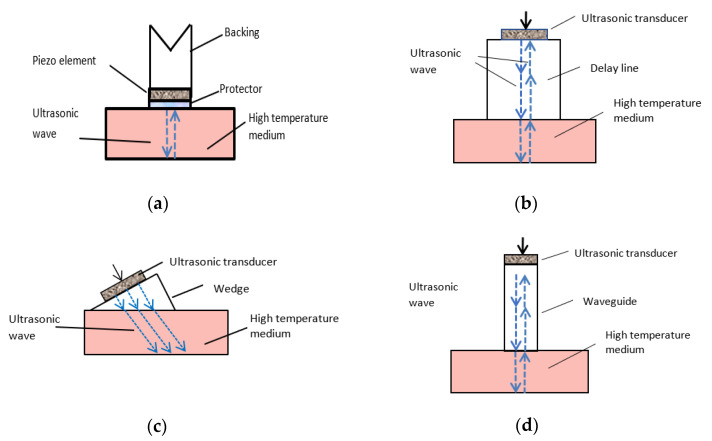
Ultrasonic transducers for extreme conditions: (**a**) with a thin protective layer; (**b**) with a delay line; (**c**) with a wedge; (**d**) with a waveguide.

Schematic images of such transducers are shown in Figure 1.

High temperature ultrasonic transducers possess piezoelectric elements and passive parts (housing, protective/matching layers, electrodes, backing materials, adhesives, and connecting wires), which should be resistant to a high temperature and protected from a medium which can be highly corrosive. For protection, all elements of a transducer are placed into a temperature and corrosion resistant housing, usually made of stainless steel. Piezoelectric elements are made of piezoelectric materials with Curie temperatures higher than required by a specific application [26]. The piezoelectric element is protected from the medium into which ultrasonic waves are radiated or from which they picked up by a thin or thick solid layer or rod.

In the case of a thin protective layer, it is usually made of stainless steel or metals with similar features. The layer protects the piezoelectric element from corrosion and mechanical impacts, but the temperature inside the transducer housing is the same as of the high temperature medium. It means that the used piezoelectric materials must possess the high Curie temperature at which piezoelectric properties are lost. If the thickness of the layer and its acoustic impedance are properly selected then it can also serve as an acoustic matching layer improving sensitivity and bandwidth of the transducer. In order to get the necessary bandwidth of the transducer and a short pulse response the piezoelectric element is damped by a backing attached to its back surface (Figure 1a). For high temperature transducers the backing is made of various metals and high temperature ceramics possessing the necessary attenuation of ultrasonic waves.

In the case of a thick solid protective element, such as a delay line or waveguide, it usually it serves not only for separation of the piezoelectric element from the environment under extreme conditions, but also to reduce the temperature of the piezoelectric element. Such protective elements can be of three types: the delay lines, wedges, and waveguides. The main difference between them is in lateral dimensions. In the case of delay lines, the lateral dimension is much bigger than the wavelength of the ultrasonic wave. The transducers with a thick protector are pressure robust. The backing usually is not needed because the piezoelectric element is damped by a medium, for example, solid or liquid metals, into which ultrasonic waves are radiated. The duration of radiated ultrasonic pulse may be just a few periods that are good for NDT and imaging purposes.

In the case of a waveguide, lateral dimension/s are comparable with the wavelength. The wedges are usually made of materials with a lower ultrasound velocity and are exploited for transformation of a longitudinal wave in the wedge into a shear wave in steel. The transducers with wedges are used for NDT of solid materials. It is necessary to point out that such thick protective elements can be made of metals such as stainless steel, titanium, quartz, or ceramics and even high temperature plastics with a low thermal conductivity. High temperature plastics such as polyimide can be used for wedges at temperatures up to 250 °C.

In some cases, ultrasonic transducers must be placed far away from electronic units used for generation of excitation signals and amplification of received ultrasonic signals. For that, quite long heavy-duty electronic cables reaching up to a few tens of meters are used that unavoidably can distort analogue type electric signals. To avoid this problem some electronic units such as power stages of the excitation generator and preamplifiers are placed inside the housing of the ultrasonic transducer close to a piezoelectric element. Usually for that, a so called system-on-chip is used in which the power unit or the preamplifiers are integrated into a specially developed chip. Considering that an ultrasonic transducer can be at an elevated temperature and no efficient cooling of the chip inside the housing can be provided, there is a danger that built-in electronic units may be overheated. To avoid this problem a small size semiconductor pn-junction-type temperature sensor is incorporated into the chip and when the critical temperature is achieved the power supply to the power unit is disconnected. For sensing temperature, a bipolar transistor is used which gives a temperature dependent voltage linearity that is better than that of thermocouple. A drawback of the ultrasonic transducers with such built-in electronic units is that the maximum operating temperature is 150–180 °C [27].

### 2.1. Ultrasonic Transducers with Matching Layers

In the case of thin protectors, they can be used not only for protection of a piezoelectric element from a hazardous environment but for an acoustic matching purpose. An acoustic impedance of high temperature media into which ultrasonic waves are radiated as a rule is rather different from the acoustic impedance of the employed piezoelectric materials. It leads to multiple reflections from the interfaces of the piezoelectric element-protector and protector—a high temperature medium. As a result, the reflected waves interfere with each other and for this reason the performance of the transducer worsens. Various matching techniques of different acoustic impedances were analyzed by Rathod in a very detailed review paper [28]. Matching should increase efficiency and/or bandwidth of the ultrasonic transducer [29]. Usually for matching purposes, the thickness of the protector *l*_p_ is selected equal to a quarter wavelength λ/4 of ultrasonic wave in it. The ideal matching is obtained when the acoustic impedance of the protector *z_p_* is

(1) $$z_{p}=\sqrt{z_{T}z_{m}}$$

where *z_T_* is the acoustic impedance of the transducer and *z_m_* is the acoustic impedance of the high temperature medium. It is necessary to keep in mind that if there are variations of the temperature, the acoustic impedances change mainly due to changes of the ultrasound velocity in the high temperature medium and the piezoelectric material. Therefore, in principle, such ideal acoustic matching may be achieved at one specific temperature. Usually, the temperature at which ultrasonic measurements will be performed is considered.

Other requirements to the protector are the required mechanical strength, resistance to corrosion, hardness, and a thermal expansion coefficient close to the thermal expansion of the piezoelectric element [30]. The last requirement is very important for high temperature transducers because if thermal expansions of neighboring media in the transducer are too different then such phenomena as delamination or even disbonding between the protective layer and the piezoelectric element may take place. Therefore, the materials should be selected based on compatibility of their thermal expansion coefficients.

In the case of fourth generation nuclear reactors cooled by liquid sodium, lead, or lead–bismuth alloy there is a requirement to ensure wettability of the protector by those liquid metals. If this requirement is not fulfilled then radiation and reception of ultrasonic waves through a protective layer can significantly diminish and ultrasonic measurements will be impossible. To find a material corresponding to such different requirements is not an easy task, therefore ranking according to priorities should be used.

Let us analyze an example of the ultrasonic transducer for operation in a liquid metal, for example, molten lead–bismuth used in fourth generation nuclear reactors. Its acoustic impedance is *z_m_* = 18 MRayl. The acoustic impedance of a majority of piezoelectric materials is around *z*_T_ = 30 MRayl. From Equation (1) it follows that for such acoustic impedance values, the optimal impedance of the protector should be *z_p_* = 23 MRayl. The acoustic impedance of the most popular stainless steel AISI 316 used for nuclear applications is 45 MRayl, what does not correspond to the optimal value required by Equation (1). It means that to achieve acoustic matching by a λ/4 thickness metallic protector in most cases is unrealistic; therefore, in many cases thickness of the protective layer is selected equal to a half wavelength λ/2. Such a protector does not provide an acoustic matching but mechanically is more robust because it is thicker. Such a transducer was proposed by Mrasek et al. [31]. The protector’s thickness was 0.6 mm, which corresponds to λ/2 at 5 MHz and it was made of Inconel-600. The transducer with a LiNbO_3_ piezoelectric element was suitable for operation up to 800 °C. Another example is the ultrasonic transducer where a piezoelectric material rhombohedral 36° Y-cut lithium niobate LiNbO_3_ was used. The protector was made of stainless-steel 321. This transducer was confirmed to work up to 800 °C when the temperature was increased with the speed 4 °C/min [32].

For additional protection of the transducer faceplate, it can be coated by a diamond-like carbon thin layer [5,30]. Such a coating also improves acoustic coupling between ultrasonic transducers and a heavy liquid metal. The long-lasting experiments, up to a few months, carried out in a liquid lead–bismuth environment in the temperature range 300–350 °C demonstrated a very good performance of such coating.

According to Amini et al., black alumina could be used as a matching layer for the transducer suitable for a high temperature environment because of its thermal stability up to 800 °C. In the transducer 36° Y-cut LiNbO_3_ piezoelectric element with the Curie temperature of 1200 °C and a 3 MHz central frequency was used. Black alumina possessing an acoustic impedance 36 MRayls was chosen because of its stability at a high temperature environment and resistance to oxidation. Its coefficient of thermal expansion 8.5 μm/m °C is similar to the coefficient of thermal expansion of LiNbO_3_, which is between 10.3 and 15.5 μm/m °C. It allows getting a stable connection among layers in a wide temperature range and to achieve a reliable operation of the transducer. This transducer was able to produce clear echo signals up to 800 °C and to maintain full functionality after cooling to room temperature [33].

As it was mentioned above, one of the most important requirements for the high temperature ultrasonic transducers used in hot liquid sodium is wettability of the protective layer. Such a property possesses a layer made of nickel that is widely used in many applications [34,35,36]. The shortcoming of this material is that the acoustic impedance of nickel is very high (50.7 MRayl) and in spite of the quarter wavelength thickness of the protective layer there is a significant mismatch of acoustic impedances between the transducer and liquid sodium. Due to that, a lower amplitude of the received ultrasonic signal is obtained. The proposed transducer was tested in water and silicon oil up to 142 °C [34]. The presented example shows that due to other priorities other than providing good acoustic matching, it is necessary to sacrifice the efficiency of an ultrasonic transducer.

### 2.2. Ultrasonic Transducers with Delay Lines

Delay lines between an ultrasonic transducer and a high temperature medium are used to reduce the temperature of piezoelectric elements and for some applications to increase the delay time of reflected ultrasonic signals [12,37,38]. The lateral dimensions of the delay lines are limited and usually they are made as buffer rods with a circular or rectangular symmetry. Lateral dimensions of the buffer rods are bigger than a wavelength of an ultrasonic wave. In rods, usually the longitudinal waves are excited. Materials from which the buffer rods are manufactured are selected according to particular applications and temperature range (Table 1).

It is necessary to point out that most of the presented parameters are temperature dependent, especially ultrasound velocity; therefore, in Table 1 their values are given at a room temperature. Velocities of longitudinal waves in some materials in temperature range up to 200 °C are presented in Table 2 [5].

The temperature dependence of an ultrasound velocity in solids may be approximate by a linear relation

*c*(*T*) = *c*_0_ − *T*_K_ ∗ ∆*T* (2)

where *c*(*T*) is the ultrasound wave velocity in a buffer rod at the temperature *T*, *c*_0_ is the velocity at room temperature *T*_0_ = 21 °C, Δ*T* = *T* − *T*_0_ is the temperature variation in a material, and *T*_K_ = (*c*_0_ − *c*(*T*))/Δ*T*) is temperature coefficient of the ultrasound velocity which shows how much this velocity changes per 1 °C. For longitudinal waves, the temperature coefficient for the stainless steel AISI 316 is *T*_K_ = 0.7 m/s/°C [37] and for titanium *T*_K_ = 2.5 m/s/°C [38].

**Figure 2:**
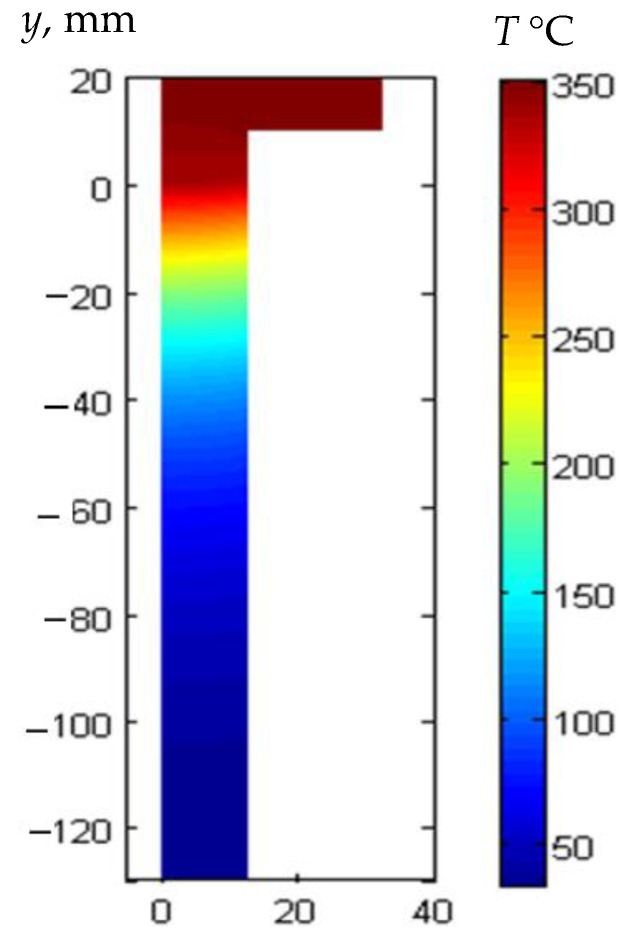
Temperature distribution along the buffer rod made of glass ceramic ZERODUR.

In a temperature range up to 300 °C, high temperature plastic materials such as polybenzimidazole (PBI) and polyimide (PI) can be used [5,39,40,41]. They are very good heat insulators and may be used for a prolonged time without forced cooling. Glass ceramic materials can also be used at higher temperatures. For example, the material ZERODUR K20 manufactured by Advanced Optics SCHOTT in Germany possesses a very low expansion coefficient 2.4 × 10^−6^ /K and may be used up to 850 °C. Temperature distribution in a buffer rod made of ZERODUR K20 ceramics is shown in Figure 2.

The length of the buffer rod was 130 mm, and the upper tip was heated up to 350 °C. Around the rod, the air was room temperature. From the presented example, it follows that a rather short buffer rod made of glass ceramic enables to reduce the temperature of the opposite tip to which the ultrasonic transducer is bonded down to 34 °C. In the case of PBI waveguide, due to a lower thermal conductivity such temperature can be achieved with the shorter 70 mm rod. It means that excitation and reception of ultrasonic signals can be performed by a conventional ultrasonic transducer.

As delay lines buffer rods made of various metals, such as steel, titanium, etc., are used. The most popular are rods made of steel due to a good mechanical strength and operation temperature up to 1200 °C. However, for not-extremely-high temperatures, up to 500 °C, aluminum (Al) rods are also used. Transducers with such rods were applied for monitoring of a process control, for example in the food and polymer industries [42,43,44].

In rods, usually longitudinal waves are excited. Propagation of such waves in rods with rectangular and circular cross-sections was investigated and analysis of trailing waves propagating slower than the longitudinal waves was performed [44]. It was found that level of the trailing waves depends on geometry of the ultrasonic transducer attached to the rod. The best performance in terms of the useful signal to the noise caused by trailing waves is obtained with the circular rod excited by the ultrasonic transducer with rectangular aperture.

**Figure 3:**
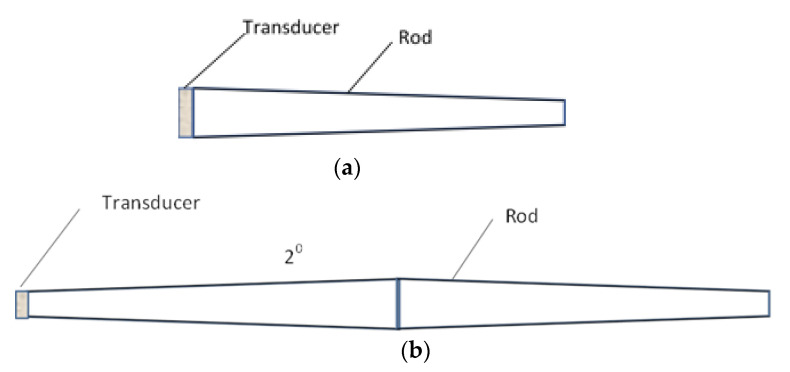
Buffer rods with circular cross-section for reducing trailing waves; (**a**) tapered rod; (**b**) double tapered.

In order to avoid additional ultrasonic waves trailing the main pulse used for measurements, clad buffer rods were proposed [45,46]. Such rods consist of a mild steel core that is coated by a stainless-steel cladding using thermal spray technology. The trailing waves can be additionally reduced by employing not cylindrical rods but tapered rods (Figure 3a) or with a double taper shape (Figure 3b). In this way, the influence of the mode converted ultrasonic waves reflected by the rod surface is reduced and the signal to the noise ratio is improved.

For measurements and imaging in melted Al using 10 MHz ultrasonic waves, the length of the buffer rod was 276 mm. For imaging purposes, the tip of the buffer rod immersed in melted aluminum was made hemispherical, thus obtaining a focused ultrasonic beam and to create a good quality image of the character engraved on a steel plate at a temperature of 780 °C [46].

**Figure 4:**
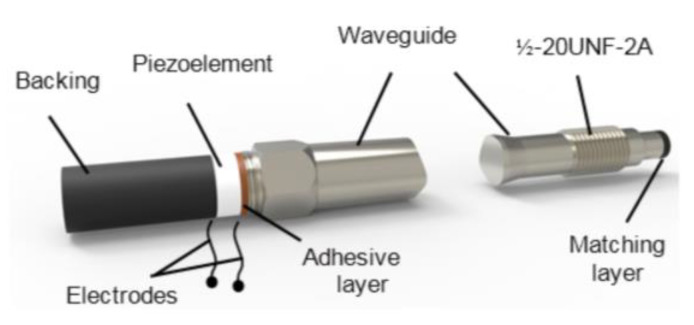
Ultrasonic transducer with a tapered buffer rod for a process control.

In the case of monitoring of high temperature substances such as melted plastics, the buffer rod must contact the liquid plastic flowing inside a high-pressure pipe [5,47]. For that the rod is screwed via the standard port ½-20UNF-2A into a wall of the pipe. Considering that for reduction of trailing waves the shape of the rod is tapered to the diameter of the smallest rod tip, defined by the diameter of the standard port, which in this case is 8 mm. Propagation of ultrasonic waves in such rod was analyzed in [48,49]. The buffer rod is made of stainless steel AISI 316 or titanium Gr.2. The image of such high temperature transducer with the tapered rod suitable for insertion via standard port is shown in Figure 4.

The length of the metallic rod is 145 mm what is enough to reduce the temperature at the front tip *T* = 200 °C down to *T* = 50 °C at the opposite end of the buffer rod. It means that in this case a standard PZT piezoelectric element such as Pz 29 with the Curie temperature *T*_C_ = 235 °C can be used. It is necessary to point out that there is no forced cooling—the cooling is due to free air convection around the metallic rod. The proposed design of the transducer enables to perform measurements not only in high temperature substances but at a high pressure also.

Performance of the transducer with a metallic buffer rod may be improved by coating the tip of the rod by a matching layer. It was found that when the buffer rod contacts the liquid polymer melt such as polypropylene, the λ/4 matching layer made of glass enamel shows the best performance in the temperature range up to 220 °C. The acoustic impedance of glass enamel is 13 MRayl and the maximum operation temperature is 1500 °C [5].

**Figure 5:**
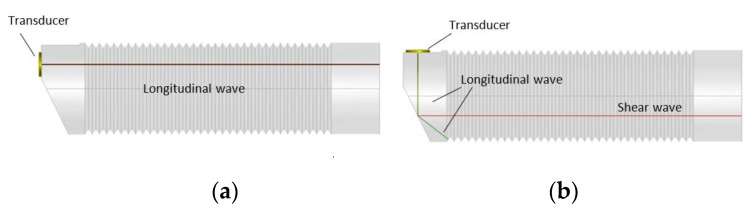
Ultrasonic transducers with threaded buffer rods: (**a**) with longitudinal waves; (**b**) with transformation of longitudinal waves to shear waves.

When measurements are performed in liquid lead–bismuth (Pb/Bi) eutectic the transducers with steel buffer rods protect not only from high temperature but from an aggressive behavior of the liquid Pb/Bi alloy also [30]. Using such transducers dependences of the ultrasound velocity in the Pb/Bi alloy versus temperature in the range from 160 °C to 460 °C for the first time were measured [50]. Influence of trailing waves was reduced by applying not tapered but cylindrical threaded rods with grooves made on an outer surface of the rod (Figure 5) [51].

The transducers with buffer rods shown in Figure 5 are of two different types. In the transducer presented in Figure 5a, a longitudinal wave is directly generated by the thickness mode piezoelectric element attached to the end of the buffer rod. For some high temperature measurements, for example for thickness monitoring, shear waves are applied. The second type of the transducer can be used for such purpose. The shear wave is obtained after reflection of the longitudinal wave from the inclined surface of the tip of the buffer rod (Figure 5b). An advantage of the latter design is that for excitation of the shear wave a conventional longitudinal mode transducer can be used. Influence of temperature gradients on propagation of longitudinal and shear waves in the buffer rods was analyzed by us in [51].

The Olympus-Panametrics Company is selling ultrasonic transducers with cylindrical delay lines suitable for high temperature operation [52]. The proposed delay lines may be used for temperatures up to 175 °C (type WTD), 260 °C (type HTD), and 480 °C (type WHTD), however not for longer than 10s. After that, the transducer with the delay line must be cooled in air at least one minute in order to avoid over-heating the transducer.

The same manufacturer of ultrasonic transducers is proposing wedges for elevated temperatures [52] suitable for excitation of shear ultrasonic waves in metals what is necessary for high temperature non-destructive testing. For example, the Olympus-Panametrics Company manufactures high temperature wedges that can be used for temperatures up to 260 °C (type ABWHT) and 480 °C (type ABWVHT); however, they do not reveal what materials are used in such delay lines or wedges. It is possible to assume that in order to get transformation of longitudinal waves to shear wave in steel for this purpose high temperature plastic materials should be used.

### 2.3. Ultrasonic Transducers with Waveguides

In the case when the object’s temperature is much higher than room temperature for measurements as delay lines long waveguides are used (Figure 1d). In this case the temperature of a waveguide tip to which an ultrasonic transducer is bonded due convection cooling by ambient air is much lower than of the high temperature object. Reduction of the temperature depends on the material from which the waveguide is manufactured, cross-section geometry, and its length. In such way it is possible to significantly reduce the temperature of the ultrasonic transducer that allows for use of piezoelectric materials with a much lower Curie temperature, for example, such as lead zirconate titanate PZT-5A. Usually two types of waveguides are used—with a rectangular cross-section and a circular symmetry, e.g., cylindrical rods. Investigations carried out by Cegla et al. [2,53] showed that according to temperature distributions along a stainless steel SS304 waveguides of different cross-section geometry and dimensions the best performance showed a thin wire with a 0.5 mm radius and a steel strip with the rectangular cross-section 1 × 15 mm. In those cases, the tip of the waveguide was heated up to 600 °C and the temperature along the waveguides of the 0.7 m length reduced to 26 °C at the opposite tip of the waveguide to which an ultrasonic transducer was fixed. For radiation and reception of ultrasonic waves into a wall two separate wave guides were used.

**Figure 6:**
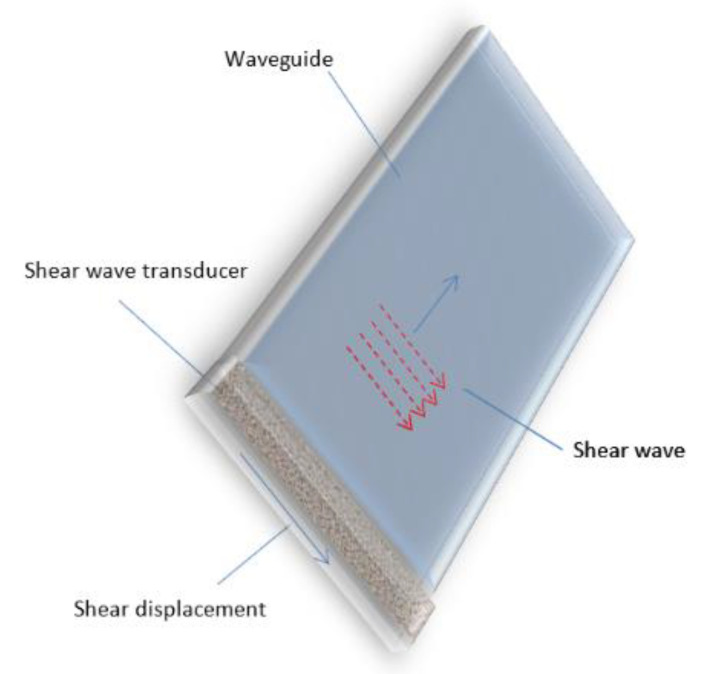
Excitation of the shear-horizontal wave in a rectangular waveguide.

It is necessary to point out that in waveguides, depending on a frequency of ultrasonic waves, geometry and dimensions of their cross-sections, different wave modes can propagate, some of which exhibit a strong dispersion. The best geometry of the waveguide is a waveguide with a rectangular cross-section [54]. In such a waveguide, a 2 MHz non-dispersive shear-horizontal wave is excited. For excitation and reception shear waves, ultrasonic transducers are exploited. Their polarization direction must be aligned parallel to the width of the strip like waveguide (Figure 6).

The advantage of such a wave is that the waveform of the ultrasonic pulse propagating in a rectangular waveguide is not distorted due to dispersion. It allows for obtaining a higher accuracy of the delay time measurements, which are used for continuous corrosion monitoring of high temperature pipes. A detailed analysis of the dispersion curves and propagation of ultrasonic pulses in rectangular waveguides was performed by us and reported in [54]. This waveguide is suitable for long term operations at high temperature, because it was tested at 730 °C for more than 4 weeks and showed good results as thickness monitoring sensor.

A similar ultrasonic high-temperature thickness monitoring system with two separate rectangular waveguides for transmission and reception of a shear horizontal ultrasonic wave was proposed and tested by Cheong et al. to monitor local thinning in carbon steel pipes caused by a flow-accelerated corrosion [4]. The length of waveguides made of carbon steel SA 106 was 300 mm. Long term measurements were performed up to 220 °C [4].

As was mentioned above, the ultrasound velocity depends on the temperature of the waveguide. This phenomenon is exploited for temperature measurements of the environment contacting the waveguide. For measurements at higher temperatures, ultrasonic waveguides are made of such materials as Al_2_O_3_, ZrO_2_, MgO, and HfO_2_, the melting temperatures of which are all higher than 2000 °C. An ultrasonic system with a waveguide made of alumina Al_2_O_3_ was used to measure viscosity of molten glass and metals up to 1500 °C [6]. Inside the waveguide, the shear horizontal wave was excited by a commercially available ultrasonic transducer. In order to keep the transducer at room temperature the waveguide was cooled by water flow.

For temperature measurement, Wei et al. also used a ceramic waveguide made of pure Al_2_O_3_ due to its resistance to high temperature (melting point 2050 °C), corrosion, and chemical stability [10]. Such a sensor can be continuously used for temperatures even above 1800 °C. The developed sensor was able to work in an oxidative high temperature environment without a protection sheath. The diameter of the waveguide was less than 1 mm diameter and the length was 600 mm. The temperature was determined from the delay time of ultrasonic pulse type signals with the central 2.5 MHz frequency reflected by the notch and the end tip of the waveguide. In this way the average velocity of a longitudinal wave in the 25 mm length sensitive segment was measured. They claimed that the ultrasonic signal amplitude remained the same while the environmental temperature was changing from 26 °C to 1600 °C.

For measurement of high temperature profiles inside furnaces and melters, two techniques with waveguides were proposed: with multiple waveguides of different length and one waveguide with a few bends [9]. The waveguides were made of Chromel, which is an alloy made of approximately 90% nickel and 10% chromium and can be used up to 1100 °C in oxidizing atmospheres. In the waveguides the longitudinal L(0, 1) mode with a conventional 0.5 MHz PZT transducer was excited. The measurement principle is based on the delay time difference between the ultrasonic signals reflected by from the bend or pair of bends and the end of waveguide. The temperature range in which measurement can be performed was from room temperature up to the temperature at which waveguide material can be used. The authors claimed that waveguides with bends were better than straight, because of a decrease of influence of the temperature gradients. The highest measured temperature limited by the used furnace was 1400 °C. The higher reliability and the ability to measure temperature profiles vertically or horizontally could be the main advantages of this method.

Applications of waveguide type ultrasonic transducers in imaging systems of sodium fast breeder reactors were analyzed in very detailed reviews [11,14].

For imaging in sodium cooled fast reactors Argonne National Laboratory, USA, developed waveguide ultrasonic transducers in which the waveguide was a bundle rod with a spiral sheet [55,56,57]. Such a design enabled reduction of trailing waves caused by dispersion and mode conversion. The length of the tested waveguides was 304.8 mm and 457.2 mm. The end of the waveguide for imaging purposes was made concaved, which allowed obtainment a focused ultrasonic beam at 5 MHz. The waveguide sensor was tested in molten sodium at the temperature 343 °C. Experimentally obtained C-scans with such transducers demonstrated ability of the system to detect defects with dimensions 0.5 × 1 mm.

Different type of ultrasonic waveguide transducers with a rectangular cross-section of stainless-steel waveguides were proposed in the UK and Korea [58,59,60,61,62,63]. The Atomic Energy Research Establishment in UK developed a novel ultrasonic waveguide transducer with the A_0_ mode Lamb wave. The length of the waveguide was 2 m and the operation frequency of the transducer was 2 MHz. The transducer was tested both in water and liquid sodium at 400 °C and showed a very good signal to the noise ratio.

Korea Atomic Energy Research Institute developed ultrasonic transducers with very long 10 m waveguides [64]. One waveguide transducer is used for transmission and another one for reception of ultrasonic A_0_ mode Lamb wave. In such a design, a leaky wave is radiated into sodium and its propagation direction can be controlled by changing the frequency of ultrasonic wave without any mechanical scanning of the ultrasonic transducer. For that, the frequency dependent phase velocity of A_0_ mode in the plate type waveguide must be higher than the velocity of ultrasonic wave in sodium 2474 m/s. That is achieved by coating the steel waveguide by a thin beryllium layer. For improvement of the wetting of the radiation zone, it is coated by a thin nickel layer. An imaging system with such transducers was used to obtain C-scan images in liquid sodium and showed a spatial resolution of 2 mm [63]. Such transducers were also applied to measure the level of molten sodium [60] and to perform imaging of various stainless-steel objects with engraved defects immersed in liquid sodium [64].

## 3. Types of Piezoelectric Materials for High Temperature Applications

In the last two decades, ultrasonic transducers used for non-destructive testing in hazardous conditions (i.e., high temperature, radiation), could be classified into six categories based on the most frequently used piezoelectric element. These piezoelectric elements are lead zirconate titanate (PZT), lithium niobate (LiNbO_3_), metaniobate, bismuth titanate (Bi_4_Ti_3_O_12_), gallium phosphate or orthophosphate (GaPO_4_), aluminum nitride (AlN), and others. The selection of a suitable piezoelectric material is based on its properties, such as the Curie temperature, thermal expansion coefficient, electromechanical coupling factor, etc.

Properties of piezoelectric materials suitable for high temperature applications are presented in Table 3.

In Table 3, the piezoelectric materials are arranged according to the Currie temperature. Aluminum nitride has the highest Curie temperature (2800 °C) of the mentioned materials, while the modified lead metaniobate and PZT has the lowest (360–570 °C). The maximal operating temperature is usually lower than the phase transition or melting temperature in order to avoid the damage of piezoelectric materials.

Each piezoelectric material will be discussed in the subsections below.

### 3.1. Aluminum Nitride (AlN)

Aluminum nitride (AlN), which is categorized as a non-ferroelectric material, could be used for high temperature applications because of its high melting point (>2000 °C) without any phase transition. AlN is known for its the Wurtzite structure, the high Curie temperature, resistance to radiation, electrical resistivity, and applications for high temperature ultrasonic transducers [65,66,67].

Furthermore, AlN is hard to obtain due to the difficulties during production. A difficulty of obtaining a thin layer of AlN, growing high-quality crystals, and weak piezoelectric properties are the main disadvantages of AlN [25,68,69].

Patel et al. distinguished the useful properties of AlN, such as high dielectric strength (>2 × 10^7^ V/cm, electrical resistivity (around 10^13^ Ω∙cm), thermal conductivity (about 200 W/m·K), and low relative dielectric constant (8.6). It was proved that an ultrasonic transducer with AlN as a piezoelectric material has high thermal conductivity, because after heat exposure (~946.85 °C), there were no cracks observed, but at a higher temperature (~1156.85 °C) an oxidation caused failure of ultrasonic response measurements [68].

Properties of AlN crystals were investigated by Kim et al. [65]. An ultrasonic transducer was manufactured with platinum films (as electrodes) and to prevent oxidation and corrosion due to high temperature, an insulator and a housing were used. A vertical tube furnace, where AlN samples were placed, was heated from 23 °C to 1020 °C. The electrical resistivity of AlN single crystals were measured at room temperature (8.8 × 10^13^ Ω·cm), at 800 °C (1.1 × 10^11^ Ω·cm), and at 1000 °C (5 × 10^10^ Ω∙cm), the results showed that these crystals had a high insulation and could maintain the long-term developed charge. For investigated resonators modes (thickness extension, length extension, thickness shear, length thickness extension, and radial), the elastic constants and the electromechanical coupling factors showed small change at thermal stability (<11.2% at room temperature and <17% at 1000 °C). The elastic, dielectric, and the piezoelectric constants of aluminum nitride single crystals had a high thermal resistivity at 1000 °C, which suggested that these crystals could be used for high temperature applications.

Tittmann et al. set several experiments with AlN and concluded that it is not the first choice for high-temperature applications due to its weak piezoelectric properties and the difficulty of growing high-quality crystals. Although, AlN piezoelectric crystals could be used for non-destructive evaluation, because after 55 h at 550 °C, at standard atmospheric conditions, the echo amplitude remained almost the same. The ultrasonic transduction of these crystals was not affected by 24 h of exposure to 950 °C and 48 h exposure to 1000 °C [25,66,67].

The high-temperature sensor for NDT was manufactured with AlN single crystal and was tested up to 800 °C and the ability to detect the Lamb waves was obtained. However, the increase of temperature caused an increase of dielectric loss [69].

The effect of irradiation was tested on AlN up to 33 MGy and almost no impact on the transfer coefficients and pulse responses was observed. Although AlN showed very good resistance to γ doses, its low electro-acoustic efficiency is the main disadvantage of choosing this piezoelectric material instead of others for application in harsh environments [21,30].

### 3.2. Oxyborate Crystals (ReCa_4_O(BO_3_)_3_)

Oxyborate crystals (a general formula is ReCa_4_O(BO_3_)_3_ are also known as ReCOB, where Re is a rare earth element, such as yttrium, erbium, lanthanum, etc. Those crystals possess high piezoelectric coefficients (3–16 pC/N) and electromechanical coupling factors (6%–31%), they are also known for not having phase transitions before the melting temperature was reached (~1500 °C). The oxyborate crystals possess high electrical resistivities (>10^6^ Ω·cm at 1000 °C) and their thermal stability (<20% variations from room temperature to ~1000 °C) allowed them to conclude that they are suitable for applications at extremely high temperatures [66,70].

Zhang et al. made an accelerometer prototype of YCOB (YCa_4_O(BO_3_)_3_) and proved it to be suitable for high temperature applications, because it showed stability up to 900 °C for 3 h with the sensitivity of 2.4 ± 0.4 pC/g in a wide frequency range (100–600 Hz) [71].

For high temperature applications, the YCa_4_O(BO_3_)_3_ piezoelectric single crystals were synthesized from CaCO_3_, Y_2_O_3_, and H_3_BO_3_, while chrome (thickness of 50 nm) and gold (thickness of 150 nm) were sputtered as the electrodes [72]. The basic properties (dielectric *ε_ij_*, piezoelectric *d_ij_*, and elastic *s_ij_* coefficients were 4, 10, and 13, respectively) of YCOB single crystals were characterized from a room temperature up to 800 °C. It was noticed that elastic compliance constants, the dielectric permittivity and loss, piezoelectric charge constants increased with the rise of temperature, while the stiffness constants, the coupling factors, and mechanical quality factor decreased. The quality factors at room temperature and 800 °C were correspondingly ~10,300 and ~1300, what means that the efficiency of the sensors should be weaker at high temperatures than at room temperature. The decrease of coupling factors (k_33_, k_15_, and k_31_ were 0.005–0.025, 0.014–0.052, and 0.008–0.046, respectively) as the temperature increases was the main disadvantage of YCOB crystals. It could be concluded that according to Zu et al. those YCOB crystals did not prove to be suitable for high temperature applications [72].

Although Zu et al. showed that YCOB could not be used as piezoelectric material based on its coupling factors at a high temperature, K. Kim et al. claimed that their shear-mode piezoelectric accelerometer manufactured using YCOB was suitable for vibration sensing applications up to 1000 °C. (YXt)−30° cut YCOB single crystals were used as sensing components. The shear mode electromechanical coupling factor (0.22), the elastic compliance (1.8 × 10^−11^ m^2^/N), the piezoelectric strain constant (10 pC/N) and the piezoelectric voltage constant (0.090 Vm/N) were calculated. To prevent oxidation and corrosion of electrodes, in this accelerometer thin film electrodes and conductive adhesives on YCOB were not used. During an experiment when temperature rose from 25 °C to 1000 °C, the sensitivity of this sensor (average 5.7 pC/g) remained almost the same at the tested frequency range (80–1000 Hz) and it was similar to a calculated value at room temperature (5.5 pC/g). The average measured sensitivity during an experiment at 1000 °C for 4 h was 5.9 pC/g. The authors explained that some variations of the measured dielectric permittivity, piezoelectric constants, temperature dependences of the elastic compliance, and/or the observed noise at elevated temperatures could be due to the heated air flow in the furnace [73].

Tittmann et al. manufactured an ultrasonic transducer (with a center frequency of 10 MHz) using YCa_4_O(BO_3_)_3_ for non-destructive evaluation at high temperatures. This transducer was held at 550 °C for 55 h with standard atmospheric conditions. The echo amplitude during this experiment was noticed to remain almost the same. The heat treatment was set for 24 h of exposure to 950 °C and 48 h exposure to 1000 °C in standard atmospheric conditions. Then, the ultrasonic testing of the transducer performance was applied, which concluded that the ultrasonic transduction was not affected by the heat. Dielectric and piezoelectric properties were measured to compare the untreated and the treated YCOB (at 850 °C for 120 h in air). The loss tangent was 0.002 and 0.0016, respectively, at 10 kHz, so it decreased by 20%, while the dielectric constant decreased only by 1% (7.7 pF for not treated and 7.6 pF for treated). The transducer with YCOB crystal was found to be capable of efficient ultrasonic transduction up to 1000 °C and more suitable for high temperature applications than LiNbO_3_ crystals [66,67].

Zhang and Yu gathered several properties of YCOB, NdCOB, and GdCOB crystals. Piezoelectric coefficients (d_eff_) were 3–10 pC/N of YCOB, 11–16 pC/N of NdCOB, and 5–13 pC/N of GdCOB. The electromechanical coupling factors (k_eff_) were 6–22%, 19–31%, and 10–27%, respectively, while voltage coefficients (g_eff_) were 0.094 Vm/N, 0.12 Vm/N, and 0.109 Vm/N, respectively. Oxyborate crystals (YCOB, NdCOB, and GdCOB) might be very promising piezoelectric materials in the future by comparing their properties (piezoelectric properties, thermal stability, electrical resistivity) to the most used and new piezoelectric materials [70].

According to Tian et al. oxyborate crystals (YCOB and GdCOB) could be used in harsh environments (a high temperature and irradiation). These crystals were affected by different doses of 6 MeV Xe^23+^(10^13^−10^16^ ions/cm^2^) at 25−850 °C. Due to irradiation slight changes were noticed: the piezoelectric coefficient d_12_ changed a little, the electrical resistivity decreased, and the dielectric permittivity increased. Those changes can be explained by modifications in structural distortion and in chemical bonding [74].

### 3.3. Lithium Niobate (LiNbO_3_)

Lithium niobate is a ferroelectric material, which has a high Curie temperature of 1142–1210 °C. The electromechanical coupling coefficient depends on the orientation of a crystal cut. One of the most popular cuts is the 36° rotated Y-cut LiNbO_3_ due to its sensitivity of vibrations in the quasi-longitudinal mode and a high coupling coefficient (k_t_~0.49) (Table 3). The main disadvantage of lithium niobate is its oxygen loss at elevated temperatures [21,66].

An ultrasonic transducer manufactured using 36° Y-cut LiNbO_3_ crystals and a stainless-steel waveguide was tested in a liquid Pb/Bi tank. This experiment was set at 350 °C for long-term continuous operation (300 h) and clear signals were still received. This transducer was also tested with γ doses up to 33 MGy. The LiNbO_3_ transducer was not affected by irradiation, because the transfer coefficients and pulse responses remained almost constant [21,30].

Lithium niobate could be used at high temperature applications (≥500 °C) due to its high Curie temperature and good piezoelectric properties. An electrochemical impedance spectroscopy resonance method was used to determine the material properties of LiNbO_3_ at high temperature (up to 750 °C), in the frequency range was from 100 kHz to 7 MHz. The degradation of LiNbO_3_ was caused by ionic conductivity or vacancy diffusion, but that was not significant. It could be concluded that lithium niobate was suitable for high temperature and high frequencies applications [77].

Mohimi et al. manufactured an ultrasonic guided wave transducer with four 41° rotated X-cut non-stoichiometric shear LiNbO_3_ (50% Li_2_O: 50% Nb_2_O_5_) crystals with dimensions 13 × 3 × 0.5 mm), which were coated with gold layers on both sides [78]. For high temperature ultrasonic guided wave testing, a lithium niobate crystal was bonded to a backing block, which was made of an aluminum oxide, using a high temperature joining technique to prevent the reverberating signals. A high temperature conductive adhesive was used to bond LiNbO_3_ electrode and fiber glass insulated pure nickel wire, which was routed through the backing block. For transmission of ultrasonic waves to or from the object under a test a waveguide made of a square steel rod (1.5 m long) was used. It was proved that for continuous monitoring of steam lines at 580 °C, lithium niobate proved to be a good candidate. When temperature rose to 350 °C, only small variations in the dielectric constant values were observed, but from 350 °C to 600 °C a significant increase was noticed. The authors concluded that this ultrasonic transducer could be suitable for contact applications up to 580 °C [78].

According to Schmarje et al. although lithium niobate has a high Curie temperature, it has low electromechanical coupling coefficients if other cuts, for example a Z-cut, are used [79]. The electromechanical coupling coefficient of this cut for a longitudinal mode is only k_t_ = 0.162. It is one of the main disadvantages to use this cut of a piezoelectric LiNbO_3_ element in ultrasonic transducers for a non-destructive testing. The Z-cut is attractive because its piezoelectric properties are more similar to an isotropic material and enables to excite a pure longitudinal mode. It is possible that 1–3 connectivity LiNbO_3_ composites, if they were fabricated by the dice and fill method, could be a proper active material in ultrasonic transducers at high temperature. In this experimental setup a Z-cut LiNbO_3_ was used as a piezoelectric material with two passive filler materials (room temperature vulcanizing (RTV) sealant and cement matrices), which had suitable properties for the operation conditions. RTV sealant, which was flexible, was used at temperatures up to 350 °C, and a high temperature cement, which was rigid, up to 1600 °C. The sensitivity and suitability for imaging of lithium niobate cement composites were tested with elements of 1 × 0.25 mm, which were configured as an array, at room temperature. Experiments showed the ability of RTV composites to transmit and receive ultrasonic signals, however poor acoustic coupling and not a high signal to the noise ratio was noticed. Those problems could be solved by using different fabrication methods. Despite that, echoes from the back wall of a 37.5 mm thick steel block were detected up to 180 °C. Using the same experimental setup, the cement composite transmitted and received signals up to 360 °C. It could be concluded that these lithium niobate composites could be used as the active elements at high temperature (up to 360 °C), because RTV and cement composites did not show any degradation after several thermal cycles [79].

The reliability of aging power plants needs to be tested by structural health monitoring techniques under working conditions [80]. To construct a high temperature ultrasonic transducer, a Z-cut LiNbO_3_ single crystal (thermal expansion coefficient 15.4 × 10^−6^/°C) was used as a piezoelectric element, a gold sputter layers with the thickness of 200 nm were placed on the top and bottom surfaces as an electrode using a high temperature ceramic adhesive. In order to reduce a thermal stress, austenite stainless steel (thermal expansion coefficient 16.5 × 10^−6^/°C) was chosen as the substrate material. Using a silver conductive paste, a high temperature mineral insulated cable was connected to the top electrode. In this experiment the transducer was placed into a quartz tube at the center of an electric furnace and heated. Multiple ultrasound echoes from the substrate remained stable from room temperature up to ~1000 °C, which proved that this ultrasonic transducer can be used for applications at very high temperatures [80].

Kirk et al. investigated parameters of LiNbO_3_ piezocomposites with an epoxy matrix at room temperature, electromechanical parameters of LiNbO_3_ piezocomposites with a cement matrix and a fabrication of an ultrasonic transducer, which should be suitable for defect detection at 400 °C [81]. The Y/36°-cut LiNbO_3_ 1–3 piezocomposites with the thickness of 4 mm were made with the Araldite hard-setting epoxy filler, which should reduce the occurrence of unwanted resonant modes around the operation frequency. The electromechanical coupling coefficients k_t_ from the finite element modelling and experiments at room temperature were 10–20% of the standard value for the bulk Y/36°-cut LiNbO_3_, which was 0.49. Instead of epoxy filler, alumina cement and zirconia cement were used to select a more suitable passive material for high temperature applications. The alumina cement degraded in water, but the zirconia cement was suitable for further experiments. During the heating of the piezocomposite up to 500 °C, the electromechanical coupling coefficient remained almost constant within 3%. In further experiments, the LiNbO_3_ /zirconia cement composite was heated in cycles to 100, 200, 300, 400, and 500 °C, after each cycle the electromechanical coupling coefficient was measured. A large reduction of the electromechanical coupling coefficient was obtained after cooling this piezocomposite from 300 °C to room temperature due to cracking of LiNbO_3_, which could be caused by a pyroelectric effect. During the last experiment, the transducer without a matching layer, which was manufactured with lithium niobate and zirconia cement, was used to detect a side drilled hole in the block at 400 °C. Impedance measurements were used to characterize this piezocomposite, the thickness mode coupling coefficient was ~0.50. The authors concluded that this lithium niobate piezocomposite could be used for thickness measurement and perhaps for defect detection, because after multiple thermal cycles there was no deterioration [81].

Another integrated ultrasonic transducer was manufactured exploiting the LiNbO_3_/PZT composite film (thickness of 125 μm), which was fabricated by the sol–gel method [82]. The center frequency and the 6 dB bandwidth were measured (4.4 and 3.2 MHz, respectively) at 800 °C in a pulse-echo mode. After five thermal cycles from room temperature to 800 °C (duration of each cycle −40 min), no deterioration of the ultrasonic performance was noticed [82].

### 3.4. Gallium Orthophosphate (GaPO_4_)

Gallium orthophosphate could be named as α-quartz analogue, but it is not a ferroelectric material as quartz is. GaPO_4_ does not have the Curie temperature, but it has a phase transition temperature which is 970 °C (Table 3). The main disadvantage of this material is a rather low electromechanical coupling coefficient which is only k_t_ = 0.15 [21,70].

We have tested properties of GaPO_4_ at high temperatures and its resistivity to radiation by γ rays. We found that this piezoelectric material had a good thermal stability up to 450 °C. The manufactured ultrasonic transducers with GaPO_4_ were tested in a liquid lead–bismuth environment and showed ability to be used at elevated temperatures but with a lower by one order efficiency than with LiNbO_3_ and bismuth titanate piezoelectric elements. Additionally, it was noticed that irradiation by a dose 22.7 MGy caused the decrease in the efficiency of the GaPO_4_ transducer by 13% [21,85].

Dhutti et al. wrote an article about a high temperature piezoelectric wafer GaPO_4_ active sensor for structural health monitoring of high temperature pipelines using ultrasonic guided waves. To produce torsional guided wave mode, *y*-cut (YXl)0° configuration gallium phosphate was selected because of its high electrical resistivity, no pyroelectricity, low acoustic losses, and good thermal properties up to 700 °C. One-hundred nanometer thick platinum electrodes were selected for this transducer, because of its conductive properties, resistance to oxidation, and thermal stability up to 650 °C. Platina and gallium phosphate had similar coefficients of thermal expansion (9 × 10^−6^/°C and 12.78 × 10^−6^/°C, respectively) so an additional matching layer was not required. The variation in the properties for the estimation of the transducer figure of merits was monitored at 600 °C for 1000 h, while the electromechanical coupling factor and elastic constants remained almost the same with variations of less than 1% and 1.5%, respectively. Thermal aging experiments and a stable thickness-shear mode response showed that this ultrasonic transducer could be used at high temperatures up to 600 °C for ultrasonic guided wave applications to superheated steam lines [86].

In another article, an ultrasonic transducer, which was manufactured with single gallium orthophosphate crystal, was used for non-destructive testing and monitoring in power plants at high temperature (up to 580 °C). For this application, piezoelectric material was coated with a platinum layer (thickness of 100 nm) as an electrode and was coupled to a carbon steel block. The 3.5 MHz transducer allowed detect a side-drilled hole with the diameter of 0.8 mm up to 580 °C, while the signal to noise ratio remained above 6 dB [87].

### 3.5. Bismuth Compounds

Bismuth titanate Bi_4_Ti_3_O_12_ has the Aurivillius structure and belongs to ferroelectrics [70]. Piezoelectric elements made of this material are commercially available. For example, the bismuth titanate piezoceramic Pz48 manufactured by MEGGITT Company (Kvistgaard, Denmark) possesses the Curie temperature 770 °C and the recommended operation temperature range up to 650 °C [88], but its electro-mechanical coupling coefficient is quite low—only k_t_ = 0.20 (Table 3). Despite this shortcoming, our investigations have shown that it is one of the best piezoelectric materials for high temperature applications. Ultrasonic transducers manufactured using Pz46 [89] type piezoelectric thickness mode elements were tested at high temperature (up to 450 °C) and long-term continuous operation up to 1000 h in the liquid lead–bismuth environment. This material is resistant to γ and neutron radiation. The transfer coefficient of the ultrasonic transducer with a bismuth titanate element decreased only by 4% after exposure to total γ doses up to 33 MGy radiation. It was proved that this piezoelectric ceramic is suitable for applications in extreme environments (high temperature, irradiation) [21,30].

The properties of the bismuth titanate elements may be improved manufacturing piezoelectric composites with supplements of other piezoelectric materials. For example, Tittmann et al. concluded that PZT/Bi_4_Ti_3_O_12_ and Bi_4_Ti_3_O_12_/LiNbO_3_ composites could work at high temperatures of 675 °C and 1000 °C, respectively [25].

The modified potassium bismuth titanate–bismuth ferrite lead titanate (KBT-BF-PT) material should be suitable as a piezoelectric material for ultrasonic transducer up to 380 °C [90]. Due to its high electromechanical coupling factors (k_t_ = 0.5 and k_p_ = 0.36 at room temperature) and the coefficient of thermal expansion (8 × 10^−6^ K^−1^), KBT-BF-PT could be used in conjunction with stainless steel or titanium materials exploited as other parts of an ultrasonic transducer [90].

Burrows et al. manufactured air-coupled flexural transducers for high temperature applications [91]. Using a high energy ball milling technique, the doped bismuth titanate powder was mixed with oxide precursors, a suitable binder and diluent to make a pseudoplastic paste. This 25 μm thick active layer (Bi_4_Ti_2.98_Nb_0.0l_Ta_0.002_Sb_0.008_O_12.02_) was placed onto a stainless-steel sheet. A platinum paste electrode was placed onto the top surface, to which a platinum wire was connected. A brass fitting with a ceramic insert was used to isolate the platinum wire lead. The air-coupled flexural transducer could operate at high temperature up to 500 °C, because of the high Curie temperature of the piezoelectric active element, which was determined through differential thermal analysis (665 ± 5 °C) and conductance measurements (675–680 ± 5 °C) [91].

### 3.6. Lead Meta-Niobate (PbNb_2_O_6_)

Lead meta-niobate (PbNb_2_O_6_) is a piezoelectric material with the tungsten bronze structure and is suitable for high temperature applications due to its high Curie temperature (>570 °C) [70]. Now it is commercially available [92]. Such materials as K-81 and K-91 manufactured by Piezo Technologies company (Indianapolis, USA) have the Curie temperature 460 °C.

Almost pure PbNb_2_O_6_ is made of PbO and Nb_2_O_5_. Properties of lead meta-niobate were investigated by Chakraborty et al. [94,95]. After several experiments (X-ray Diffraction (XRD) Rietveld analysis, Fourier Transform Infra-Red spectroscopy (FTIR), and Scanning Electron Microscopy (SEM)) a meta-stable orthorhombic crystallographic structure was established, which should be polarizable and ferroelectric at high temperature. An impedance spectroscopy was used to determine an electrical permittivity, which showed the highest peaks at 580 °C at 5.5 MHz and at 573 °C at 20 Hz [94,95].

There were some attempts to improve the piezoelectric properties and to increase the Curie temperature of lead meta-niobate by several dopants which were mixed into lead meta niobate but in most cases, they did not achieve any significant results.

For example, Sahini et al. investigated pure PbNb_2_O_6_ and its substitutions with Ca_2_Ti_2_O_6_, BiTiNbO_6_, Bi_0.5_Na_0.5_Nb_2_O_6_, and Bi_0.5_K_0.5_Nb_2_O_6_. Comparing dielectric, electric, and piezoelectric properties, pure PbNb_2_O_6_ had better results than its substitutions, because an increase of substitution level caused a decrease in all of those properties. Only a substitution with Ca_2_Ti_2_O_6_ (2%) showed almost the same results as pure PbNb_2_O_6_ [96].

Yue-Ming et al. used calcium and titanium as dopants in PbNb_2_O_6_. The new type of ceramic was obtained as (1 − x)PbNb_2_O_6_–xCa_0.6_TiO_3_ (x = 0, 0.01, 0.02, 0.03, and 0.04 mol). The increase in a concentration of dopants increased the Curie temperature from 565 °C to 612 °C, but it decreased the piezoelectric constant from 81 pC/N to 69 pC/N, the quality factor from 26.9 to 19.6, and the electromechanical coupling factor from 0.374 to 0.331 [97].

Pb_0.97_Ca_0.03_Nb_2_O_6_ + xwt%CeO_2_ (x = 0, 0.3) composites were made and then tested as piezoelectric materials. In this experiment the decrease of the Curie temperature was noticed from 542 °C (when x = 0) to 526 °C (when x = 0.3) as it did in k_p_ (from 0.10 (x = 0) to 0.06 (x = 0.3)). However, the piezoelectric constant d_33_ increased from 52 pC/N to 71 pC/N as well as electromechanical coupling coefficient k_t_ from 0.35 to 0.40, when the concentration was changed from x = 0 to x = 0.3 [98].

### 3.7. Lead Zirconate Titanate (PZT)

Lead zirconate titanate piezoelectric material Pb(Zr,Ti)O_3_, known as PZT, has a Perovskite structure and belongs to ferroelectric ceramics. Now it is commercially available and manufactured by many companies. As an example, the Meggitt Company (Kvistgaard, Denmark) manufactures piezoelectric elements with relatively high Curie temperatures (Table 4) [99]. Those elements possess good piezoelectric properties (d_33_ > 330–575 pC/N) and high electromechanical coupling factors (k_33_ > 70%) [99].

With increasing of temperature, the relative dielectric permittivity of those elements is also increasing from 1800 up to approximately 3000. It means that electric capacitance of such transducers at higher temperatures will be higher than at room temperature. It may influence operation conditions of an electric generator used for excitation of such transducers and affect performance of electric matching circuits if they are used.

Ultrasonic transducers with PZT type piezoelectric elements are already used for years in harsh conditions, including nuclear applications. For example, they were exploited for under sodium viewing at temperatures up to 250 °C [13]. Bilgunde and Bond analyzed the possibility to use such transducers for inspection of small modular reactors by an immersion technique [100]. They tried to find out the reasons why a thermal degradation of the used ultrasonic transducers with PZT type elements takes place, which leads to a rather limited signal to noise ratio. In order to solve this problem, they applied a finite element method for simulation of pulse echo experiments in liquid sodium at two different temperatures 105 °C and 195 °C. The main reason of the low signal to noise ratio seems to be due to debonding of the PZT element caused by a thermal stress. Variations of the properties of PZT type element influence ultrasonic signals much less.

Let us analyze some non-conventional applications of transducers with PZT type elements. The ultrasonic sensor for a stress measurement was developed by Asadnia et al. using a PZT type thin-film Pb(Zr0.52Ti0.48) micro diaphragm [101]. During experimental investigations, some fluctuations of the resonant frequency were detected when the temperature rose. Those fluctuations were probably caused by changes of PZT(0.52/0.48) phase and lattice parameters, but this transducer could be still used for high temperature sensing in real-time applications [101]. The performed experiments have showed suitability of such transducer for high temperature (≤390 °C) and high pressure (≤105 kPa) applications.

### 3.8. Other Materials

There were many attempts to develop novel piezoceramic materials suitable for high temperature ultrasonic transducers. An example is the ceramic type material (1 − x) BiScO_3_ − xPbTiO_3_, where x = 0.64, which was the first investigated by Eitel et al. [102,103]. This material possesses a high Curie temperature 450 °C and demonstrates good piezoelectric properties (d_33_ > 400 pC/N).

Fei et al. proposed the ceramic 0.36BiScO_3_-0.64PbTiO_3_, which was made of Bi_2_O_3_, Sc_2_O_3_, PbO, and TiO_2_ [104]. The measured properties of the developed 0.36BS-0.64PT ceramics at room temperature were the following: the Curie temperature 447 °C, the piezoelectric constant *d*_33_ was 450 pC/N, the relative permittivity was 1253, the dielectric loss 0.039, the remnant polarization 31.7 μC/cm^2^, and the coercive field was 19.8 kV/cm. Using the resonance method, the electromechanical coupling coefficients were determined k_p_ = 0.56, k_t_ = 0.535, and *k*_33_ = 0.707. The developed piezoelectric ceramic was exploited to manufacture a high temperature ultrasonic transducer, which could be used in the temperature range up to 200 °C. Bilgunde and Bond [105] developed a high temperature ultrasonic transducer for structural health monitoring in which the piezoelectric element was made of BiScO_3_-PbTiO_3_. For investigation of the temperature influence on a performance of the transducer, they developed a finite element model that takes into account 10 materials coefficients of the piezoelectric element. In this way they have simulated temperature dependences of the dielectric permittivity, the capacitance and the electric charge in the temperature range up to 300 °C.

Experimental investigations of the transducer with a resonance frequency 2.4 MHz were performed by a pulse echo contact method at high temperature (≤260 °C). The ultrasonic signals were radiated into a low-carbon-steel specimen. It allowed evaluation not only of the performance of the piezoelectric BiScO_3_-PbTiO_3_ element, but also the quality of an acoustic coupling between the piezoelectric element and the steel specimen. A coupling issue is one of the most urgent problems at elevated temperatures, because according to the authors almost in 60% cases at such temperatures ultrasonic transducers have acoustic coupling problems. For coupling of the piezoelectric element to the steel sample, a two-part epoxy Epotek 353ND was used. For continuous operation, the maximum allowed temperature for this epoxy is 250 °C. After several experiments, it was found that the most significant reduction of the amplitude of the first received pulse echo signal reaching 6 dB is between 200 °C and 260 °C. The main conclusion of the performed research was, that it was not clear what caused the reduction of the amplitude. Very likely it was not due to degradation of the piezoelectric BiScO_3_-PbTiO_3_ element but to the epoxy used for bonding of this element to the steel sample [105].

Pb_0.92_Sr_0.08_(Nb_1-y_Ta_y_)_2_O_6_ (PSNT) (*y* = 0, 0.01, 0.03, 0.05, 0.07, 0.09) ceramics were tested to sort out the best candidate for high temperature ultrasonic transducers. It was noticed that the component with the smallest concentration of tantalum (*y* = 0.01) proved to be the most suitable candidate, because of the high Curie temperature 562 °C, the dielectric constant, which remained almost constant up to 400 °C, the piezoelectric constant (d_33_ = 79 pC/N), and the reasonable electromechanical coupling coefficient (k_t_ = 35.33%) [106].

Langasite crystals, such as Ca_3_TaAl_3_Si_2_O_14_ (CTAS) and Ca_3_TaGa_3_Si_2_O_14_ (CTGS), are not pyroelectric materials, do not have phase transition, and their melting temperatures are as high as 1200 °C–1550 °C, but operating temperature is 700–1000 °C due to its resistivity. The main parameters of CTGS at room temperature are the piezoelectric coefficient *d*_eff_ = 4.6 pC/N and the electromechanical coupling factor *k*_eff_ = 12%, while these parameters of CTAS are 4.3 pC/N and 14%, respectively. According to Zhang and Yu, these crystals could be promising candidates for high temperature applications due to its temperature stability, electrical resistivity, and piezoelectric properties [70].

Tittmann et al. mentioned some rarely used piezoelectric materials, which are strontium niobate, lanthanum titanate, neodymium titanate, calcium niobate, and praseodymium titanate. Due to the high Curie temperature (1327–1550 °C) they could be promising candidates for high temperature applications for future work, but for now there is a lack of information about these materials [25].

## 4. Bonding Methods

As it was shown in Section 2, ultrasonic high temperature transducers consist of a plate-like piezoelectric element, protector, and backing, which is used to widen the bandwidth of the transducer. All those elements must be reliably joined together in order to get a good acoustic coupling. In this case ultrasonic waves are well transmitted from the piezoelectric element through interfaces piezoelectric element-protector and piezoelectric element-backing. Such ultrasonic transducer also must be reliably coupled to a medium-liquid or solid into which or from which ultrasonic waves are transmitted or picked up. In the case of high temperatures, the following problems arise:

1. Due to different thermal expansion coefficients of contacting materials a partial or complete delamination between those elements may occur;
2. Our experience shows that adhesion between electrodes and commercially available piezoelectric elements worsens what may lead to a failure of the transducer.

It is necessary to point out that when BIT/PZT films are used as piezoelectric elements there is no bonding problem at all, because those films are deposited on a solid backing or a buffer rod [109].

All bonding techniques may be sorted into two groups: a liquid coupling and a solid bonding of transducer elements. There are two types of liquid couplants: silicon oils and glass solders [30]. Silicon oils are liquid at a room temperature, but at temperatures higher than 250 °C they evaporate. Therefore, application of silicon oils is limited by 250 °C. Contrarily the glass solders, for example NaPoLi, are solid at room temperature and start to be liquid at temperatures higher than 250 °C. However, at such temperatures the glass solder affects chemically elements of the ultrasonic transducer and cannot be used at temperature higher than 500 °C [33,110]. Most of liquid couplants are not resistant to γ and neutron radiation.

The solid bonding technologies are the following:

1. Joining with glue;
2. Soldering;
3. Brazing;
4. Dry contact and diffusion bonding.

Let us briefly discuss properties of those bonding technologies.

1. Joining with glue.

Fei et al. used a conductive adhesive, which is suitable for high temperatures (up to 500 °C), to connect an electrode to the backing material. This transducer was able to work up to 200 °C [104].

Bilgunde and Bond used commercially available conductive paste (Duralco-124) to bond an electrode and wire. According to the seller, this conductive paste is made of an epoxy resin with silver filler, it possesses a good conductivity, and it could work up to 340 °C [111]. The developed ultrasonic transducer was tested only up to 142 °C, because signal loss was obtained. Although sellers assure that this conductive paste could be used up to 340 °C, this failure could be explained by the degradation of the conductive paste, which was used to replace a solder [34,111].

Another commercially available adhesive Cotronics 989 was tested by Giurgiutiu et al. to bond several elements of various high temperature ultrasonic transducers [112]. The Cotronics 989 is an alumina-based adhesive and could work up to ~1648 °C [113]. At first, GaPO4 piezoelectric element and titanium were bonded. Clear ultrasonic signals were obtained up to 426 °C, but at higher temperatures they disappeared. The authors claimed that it could be due to the adhesive layer. Parks et al. used this adhesive to bond AlN and silicon carbide, but this time it worked up to 1000 °C for 8 h [114]. The degradation of response was explained that it could be caused by oxidation of AlN [33,113].

Disadvantages of bonding with organic glues are that they are not resistant to a radioactive radiation and possess the acoustic impedance much lower than acoustic impedances of piezoelectric elements, metallic backings, and protectors.

2. Soldering

Using this technology, the piezoelectric element is soldered to the metallic protector and the metallic backing using a solder. In this case the melting temperature of the solder must be higher than the required operation temperature of the transducer, but lower than the Curie temperature of the piezoelectric element. There are two types of solders: soft solders with the melting temperature lower than 450 °C and hard solders with the melting temperature higher than 450 °C. Joining of metallic elements by solders with melting temperatures higher than 450 °C is called brazing [115,116]. The melting temperature depends on the chemical composition of solders. Soldering by soft solders is simpler than with hard ones. As an example of the soft solder is Pb/Sn/Ag solder with a melting temperature of 310 °C.

Another commercially available solder is 88Au-12Ge consisting of 88% gold and 12% germanium, which is suitable for high temperature joining of dissimilar metals especially with different thermal expansion coefficients. It is a ductile material and possesses a thermal expansion coefficient 13.4 × 10^−6^/°C. Before soldering the surfaces of the metals should be coated by thin nickel layers (1.5–2.5 µm) protected with an extremely thin layer of gold (0.2–0.5 µm) and soldering performed in vacuum. The melting temperature is 361 °C, but the soldering temperature should be above 380 °C [115].

3. Brazing

Brazing is a technique used to bond two or more metals to each other by melting the filler that should melt at lower temperatures than metals, which should be bonded, in order not to damage them. The main advantages of brazing are the following: it is a simple method, it could manage stress and heat very well and it could be used to join metals and non-metals, as well as dissimilar metals and porous components [116,117,118].

Amini et al. applied the brazing technique for bonding of high temperature ultrasonic transducer elements [33]. For that, they used TiBranze Al-665 foil, which was placed between LiNbO_3_ piezoelectric element-matching layer and piezoelectric element-backing. The TiBrazeAl-665 foil consists of Al, Cr, and Mg. The melting temperature of it is 645 °C. For comparison they have tried also bonding by Duralco 956 and Pyro-Duct 597-A adhesives. With the adhesive Duralco 956 a very weak adhesion was obtained. The Pyro-Duct 597-A adhesive provided a mechanically good but acoustically bad contact between the piezoelectric element and the backing. However, brazing allowed obtainment of a stable bond up to 800 °C between the piezoelectric element and the quarter wavelength matching layer.

Bosyj et al. tested brazing techniques for ultrasonic transducers with LiNbO_3_ for high temperatures applications [119]. There are two types of brazing materials: active and passive. Active brazes are used to bond the parts of the transducer by chemical reaction and usually they are made of aluminum–chromium–magnesium, tin–silver–titanium, or silver–copper–titanium and showed good results in bonding ceramics. Passive brazes (e.g., gold- or silver-based alloys) are used to bond parts by adhesion and mechanical interlocking. Comparing TiBraze^®^ Al-665 (Hilliard, OH, USA), Morgan Gold-ABA^®^ (Hayward, CA, USA), Gold, and AgCu brazes and brazing techniques (vacuum brazing and reactive air brazing), the best results were obtained using TiBraze^®^ Al-665 and AgCu. The transducers, made using those brazes, were able to transmit and receive signals up to 800 °C [119].

4. Dry contact and diffusion bonding

A dry acoustic coupling is based on pressing with a high pressure of the transducer elements together at a high temperature [30,31,57,80,120]. The temperature during bonding is *T_melt_*/2, where *T_melt_* is the melting temperature of the material. In the case of a piezoelectric element the selected temperature is *T_C_*/2, where *T_C_* is the Curie temperature. The required pressure at which bonding is performed may be very high and reach up to a few hundred MPa [121,122,123]. However, at such a high pressure, cracks can develop which may damage the piezoelectric element.

In order to avoid this problem Bhadwal et al. proposed the transducer in which a spring is used to keep a constant pressure between transducer elements in a wide temperature range. The acoustic contact is obtained due to use of soft foils coupling all parts of the spring-loaded transducer at the pressure of only 25 MPa [32]. Such coupling enabled getting a good signal to noise ratio.

Applying a dry contact bonding method, diffusion bonding can be achieved. The diffusion bonding is known as a technique for joining similar or dissimilar metals during which atoms of those metals migrate into each other. In order to apply such a joining method in the case of high temperature ultrasonic transducers the contacting surfaces of a piezoelectric element, protector and backing must meet some strict requirements:

- The surfaces must be flat with waviness less than 1 µm and polished in order to achieve a surface roughness less than 0.01 µm;
- According to our experience the electrodes of commercially available piezoelectric elements should be removed before the bonding process because of a rather weak adhesion, which is failing during cyclic variations of temperature;
- New Ni electrodes by 1 μm thickness are deposited on a piezoelectric element by electroless plating. Those electrodes are additionally electroplated by Ag layer (~10 μm) to eliminate the remaining surface roughness. Finally, an additional layer of gold (Au) with thickness 2 μm is deposited by electroplating;
- The contacting surfaces of other elements of the transducer (protector, backing) are also coated by such metallic layers.

In order to avoid disbonding or delamination during exploitation, thermal expansion coefficients of bonded materials should be as close as possible, but usually it is difficult to meet such a requirement. Therefore, between bonded surfaces a thin ductile metallic foil is inserted that partially compensates stresses caused by variations of temperature due to different thermal expansion coefficients. For such a purpose, Bhadwal et al. proposed to use an annealed 50 mm thickness silver foil [32]. We have proposed to use a soft gold foil with a purity of 99.99% with a thickness 5–20 μm, which is more expensive than the silver foil, but it is more ductile [124,125]. In such way a good quality acoustic contact between a stainless-steel AISI-316 waveguide and a piezoelectric element was obtained [30]. Wang et al. used a gold foil to bond an ultrasonic transducer and a waveguide, which were tested in liquid sodium at high temperature 340 °C [57]. PZT and LiNbO_3_ type piezoelements were bonded to other parts of transducers using a silver pressure bonding in order to compare these transducers at different environments. It was decided that this coupling method was a successful for both transducers at 320 °C in liquid sodium and at 550 °C for almost one year [120].

In order to reduce the pressure required during the bonding process, Bhadwal et al. [32] used repeated three cycles loading which allowed reducing the pressure on a piezoelectric crystal down to 12.7 MPa instead of 76 MPa in the case of the first loading cycle. Such low pressure still allowed for obtaining a good quality acoustic contact.

Another method allowing reduction of the required pressure is thermo-sonic diffusion bonding [30,124,125]. Contacting surfaces of the bonded elements must be prepared in the same as in the case of a conventional diffusion bonding—they must be polished up to optical quality and coated by Ni, Ag, and Au layers, as was described above. During the thermo-sonic bonding, powerful 17 kHz ultrasonic vibrations are introduced into a bonding zone with a Langevin type ultrasonic transducer provided with a horn. Those ultrasonic vibrations create tangential displacements in the contact zone, which facilitates the bonding process. The bonding quality depends on the applied pressure and the temperature of the contact zone. Depending on the quality of contacting surfaces, the necessary pressure is only 40–60 MPa. At this optimal pressure the temperature is gradually increased and the Au foil inserted between the bonded elements is softening. The ultrasonic vibrations are introduced in the bonding zone when 100 °C temperature is achieved. The temperature is increased further up to about 200 °C.

The pressure and the temperature necessary for bonding are significantly reduced to the level acceptable for a piezoelectric element due to introduction of ultrasonic vibrations. Ultrasonic transducers manufactured using this technology successfully operated in a liquid lead–bismuth environment at elevated temperatures [30,125].

## 5. Backing Elements

Backing is a transducer element attached to the back surface of a piezoelectric element for damping its resonant vibrations and widening a frequency response. It means that the backing layer absorbs some of the ultrasonic energy radiated by a piezoelectric element. For an efficient damping, the acoustic impedance of the backing should be close to the acoustic impedance of the piezoelectric element and there should be no strong ultrasonic signals reflected by a back surface of the backing. The last requirement is fulfilled by selecting materials with a high attenuation and/or using backings not with a flat back surface. For non-destructive and medical applications at room temperature, backings are usually made of epoxy resin with various fillers such as a tungsten powder are used; however, they are not suitable for operation at elevated temperatures higher than 200 °C.

The main additional requirements to high temperature backings are the following:

- Backing material must be suitable for temperature range required by a particular application;
- In some cases, the backing must be resistant to γ and neutron radiation;
- Backing element should possess a thermal expansion coefficient similar to that of piezoelectric material;
- Acoustic properties such as the attenuation and acoustic impedance of the backing should be stable in the whole required temperature range.

**Figure 7:**
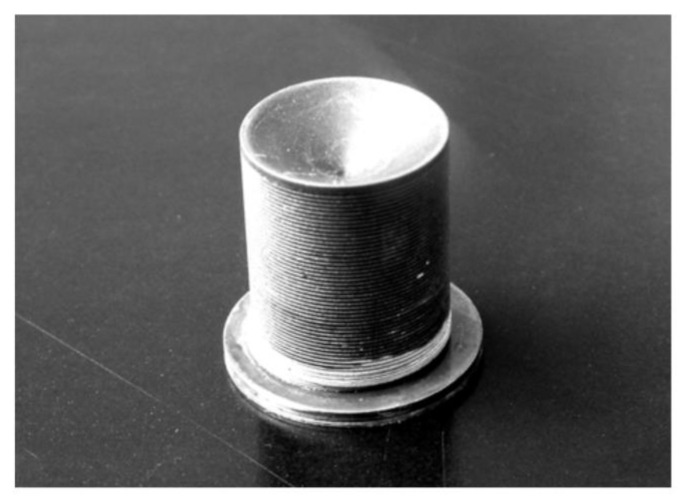
High temperature ultrasonic transducer with an integral concaved backing.

The simplest backing partially corresponding to those requirements is a backing made of stainless steel, for example AISI 316. Ultrasonic transducers with such backing possess a short pulse response [21]. However, attenuation of ultrasonic waves in AISI 316 is not very significant—only 0.13 dB/cm at 2 MHz—therefore multiple reflections inside the backing arise. In order to suppress reflections from a back surface, it is made concaved-such as a cone with a spread angle around 120° directed to the piezoelectric element (Figure 7) [21,125].

Another solution is to make the backing from a bundle of metallic wires of different length. Ultrasonic waves in such bundles propagate as in interconnected acoustic waveguides. Propagation of ultrasonic waves in similar bundles was investigated by us [126]. Due to different lengths of wires, the reflected signals return to the piezoelectric element at different time intervals and a resultant ultrasonic wave is of much lower amplitude. Such bundles may be bonded to a piezoelectric element by soldering or diffusion bonding technique.

There were many attempts to create or apply novel materials possessing a high attenuation of ultrasonic waves. Parks et al. tried a porous carbon–carbon composite as a backing element; however, they found that it was not suitable for experiments at 550 °C after 55 h due to losses of volume and mass. Then, they chose a compressed aluminum foil as a backing element which showed a good performance [66,67].

Fei et al. set an experiment with an alumina Al_2_O_3_ as a backing, but this transducer was tested only up to 200 °C [104,127].

Amini et al. proposed for backing to use porous ceramic made of 3 mol% yttria-stabilized zirconia [33,128]. The spherical pores inside material are formed introducing polyethylene microspheres into the ceramic powder. Those spheres are burned during the sintering process. They investigated attenuation of ultrasonic waves in such material in the frequency range from 1.5 to 4.5 MHz and found that depending on a pores’ size it can be from 1.5 up to 5.5 dB/cm. In this way a stable performance of the backing up to 800 °C was achieved. The acoustic impedance of the backing depends on porosity and in the range from 30% to 10% is increasing from 20 MRayl to 34 Mrayl when porosity is decreasing.

Boubenia et al. for backings proposed to use metal composites such as tin–tungsten composites with different tungsten volume fractions [129]. One metal is used as a matrix (Pb) another is used as inclusions (W). The acoustic impedance of such composites is in the range from 25 to 31 MRayl. The attenuation of ultrasonic waves is very high, from 20 dB/cm up to 191 dB/cm. The main disadvantage of such composite is that its operation temperature is lower than the tin melting temperature 232 °C.

The metal composites made of aluminum and 5% per volume tungsten may be used up to 600 °C. The attenuation of ultrasonic waves in such composite is extremely high and is increasing with a frequency. In the frequency range 3.5–6 MHz it is from 125 up to 165 dB/cm. The acoustic impedance in this frequency range is 8–10 MRayl [129].

We have proposed and tested backings made of graphite bronze [124,130,131]. The operation temperature of the graphite alloys may be up to 800 °C. Parameters of graphite bronze were the following:

- The density was ρ = 6.7 g/cm^3^;
- The ultrasound velocity was c = 2050 m/s;
- The acoustic impedance 13.7 was MRayl.

**Figure 8:**
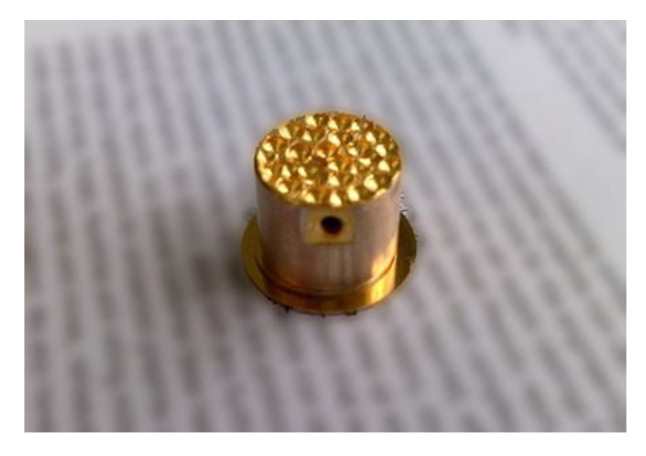
Ultrasonic transducer with a graphite bronze backing on the top.

The image of the transducer elements including the graphite bronze backing after the diffusion thermo-sonic bonding is shown in Figure 8 [125].

**Figure 9:**
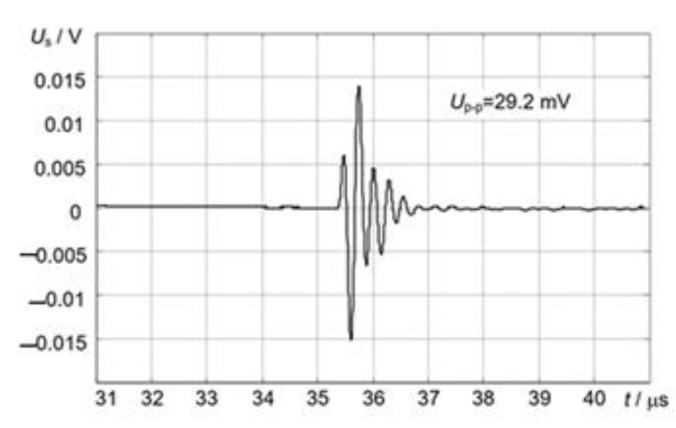
Waveform of the ultrasonic pulse obtained in a pulse echo mode in a liquid lead bismuth alloy at 290 °C.

Please note the irregular surface of the back surface of the backing in Figure 8, which enabled suppressing of the reflected back ultrasonic wave. It allowed reducing the thickness of the backing down to 10 mm. A waveform of the ultrasonic pulse obtained in a pulse echo mode in a liquid lead bismuth alloy with the 5 MHz ultrasonic transducer possessing the graphite bronze backing is shown in Figure 9. The measurements were performed at the temperature 290 °C.

Advantages of this method are a rather simple manufacturing technology of the backing, availability of the backing material, and a low cost.

## 6. Conclusions

High temperature ultrasonic transducers possess piezoelectric elements and passive parts (housing, protective/matching layers, electrodes, backing materials, adhesives, and connecting wires), which should be resistant to a high temperature and protected from a medium that can be highly corrosive. High temperature ultrasonic transducers can be of two different types: with a thin protector between a piezoelectric transducer and a high temperature medium and with a delay line or waveguide instead of the thin protector. The main difference between those two designs is that the first transducer type must operate at the same high temperature as of the medium but the transducers of the second type may operate at much lower temperatures than the temperature of the medium. In the latter case only a delay line must be resistant to a high temperature. In the case of the first type, transducers the piezoelectric elements must be made of materials suitable for operation at high temperatures. In this review, the following piezoelectric materials were analyzed: lithium niobate (LiNbO_3_), lead metaniobate, bismuth titanate (Bi_4_Ti_3_O_12_), gallium orthophosphate (GaPO_4_), aluminum nitride (AlN), lead zirconate titanate (PZT), oxyborate crystals, langasite crystals, and other materials. Aluminum nitride has the highest melting temperature (2800 °C) of the mentioned materials, while the modified lead metaniobate and PZT have the lowest (350–570 °C). One of the most popular materials is 36° rotated Y-cut LiNbO_3_, which has a high Curie temperature 1142–1210 °C and a high electromechanical coupling coefficient (k_t_~0.49). The novel materials, such as langasite crystals CTAS and CTGS, do not have phase transition and possess melting temperatures that are as high as 1200–1550 °C, but operating temperature is 700–1000 °C. In general, the maximum operation temperature is usually lower than the Curie temperature or the phase transition and the melting temperature in order to avoid damage of piezoelectric materials. Although, the highest operation temperature belongs to AlN, but other piezoelectric materials should be considered taking into account other important properties (electromechanical coupling coefficients, the piezoelectric, and dielectric coefficients) to get the best performance of the ultrasonic transducers at high temperatures.

Ultrasonic high temperature transducers consist of a plate-like piezoelectric element, protector, and backing, which are used to widen the bandwidth of the transducer. All those elements must be reliably joined together in order to get a good acoustic coupling. All bonding techniques may be sorted into two groups: a liquid coupling and a solid bonding of transducer elements. The most reliable are solid bonding technologies such as soldering, brazing, dry contact, and diffusion bonding.

The backing is used to absorb some of the ultrasonic energy radiated by a back surface of a piezoelectric element. For an efficient damping, the acoustic impedance of the backing should be close to the acoustic impedance of the piezoelectric element and there should be no strong ultrasonic signals reflected by a back surface of the backing. Selected materials should be with a high attenuation and/or using backings not with a flat back surface. For that purpose, various novel materials including porous ceramic, metal composites, and graphite bronze are used.

## Figures and Tables

**Table 1:** Materials of buffer rods and their properties.

Material	Type	Maximal Application Temperature, °C	Thermal Conductivity W/K × m	Ultrasound Velocity c_L_, m/s	Acoustic Impedance *Z*, MRayl
Polyimide (PI)	TECA-SINT 1011	300	0.22	2465.2	3.29
Polybenzimidazole (PBI)	Duratron CU60	343 (in air)	0.40	2985.3	3.85
Glass ceramic ZERODUR	ZERODUR K20	850	1.63	-	-
Aluminum (Al)	-	500	205	6320	16.9
Stainless steel	-	1200	18–20	6100	27–29
Titanium	Various alloys	1200	15–27	5740	44–48

**Table 2:** Ultrasound velocities in materials of buffer rods.

Material					
	Steel	Titanium	Aluminum Powder	Duralco	PBI
T, °C	Ultrasound Velocity c_L_, m/s				
20	5740	6203	4700	2615	2970
100	5681	6003	4593	2215	2790
150	5649	5878	4565	1980	2685
200	5614	5753	4510	1720	2575

**Table 3:** Piezoelectric materials and their properties.

Piezoelectric Material	AlN [65,66,67,68,69]	YCOB [69,70,71,72,73,74,75,76]	LiNbO_3_ 36° Y-Cut [21,77,78,79,80,81,82,83,84]	GaPO_4_ [21,85,86,87]	Bismuth Titanate, Bi_4_Ti_3_O_12_ [88,89,90,91,92,93]	Modified Lead Metaniobate PbNb_2_O_6_ [89,92,93,94,95,96,97,98]	PZT [70,99,100,101,102,103,104,105,106,107,108]
Curie temperature, °C	2800 ^1^	>1500 ^2^	1142–1210	970 ^2^	600	400–570	160–365
Maximal operating temperature, °C	1100 (With protection from oxidation)	1000	~1000 (With protection from oxidation)	700–900	<700	300	<350
Commercial availability	yes	yes	yes	yes AVL LIST GmbH	yes Piezo Technologies, FEROPERM	yes Piezo Technologies’	yes
Thermal expansion coefficient, 10^−6^/°C	20–36	-	15.4	12.78	9	1.3–1.5	3.0–3.5 ppm/°C
k_t_	0.2	-	0.49	0.15	0.23	0.33–0.43	0.49–0.55
k_26_	-	0.22	-	469		-	-
k_33_	0.391–0.395	5.1%	0.57	-	0.15	0.33–0.47	0.72–0.75
d_26_, pC/N	-	10	-	-	-	-	-
d_33_, pC/N	13.5	1.6	6	-	-	85–200	390–650 × 10^−12^ m/V
g_26_	-	0.090 Vm/N	-	-	-	-	-

^1^ Melting temperature. ^2^ Phase transition temperature.

**Table 4:** Temperature properties of PZT type piezoelectric materials.

Piezoelectric Type	Curie Temperature, °C	Recommended Highest Working Temperature, °C
Pz23	350	250
Pz27	350	250
Pz29	235	150
